# In Vitro and Clinical Safety Assessment of the Multiple W/O/W Emulsion Based on the Active Ingredients from *Rosmarinus officinalis* L., *Avena sativa* L. and *Linum usitatissimum* L.

**DOI:** 10.3390/pharmaceutics13050732

**Published:** 2021-05-16

**Authors:** Ugne Zlabiene, Juste Baranauskaite, Dalia M. Kopustinskiene, Jurga Bernatoniene

**Affiliations:** 1 Institute of Pharmaceutical Technology, Faculty of Pharmacy, Medical Academy, Lithuanian University of Health Sciences, Sukileliu pr. 13, LT-50161 Kaunas, Lithuania; ugne.cizauskaite@lsmuni.lt (U.Z.); DaliaMarija.Kopustinskiene@lsmuni.lt (D.M.K.); 2 Department of Analytical and Toxicological Chemistry, Faculty of Pharmacy, Medical Academy, Lithuanian University of Health Sciences, Sukileliu pr. 13, LT-50161 Kaunas, Lithuania; juste.baranauskaite@lsmuni.lt; 3 Department of Drug Technology and Social Pharmacy, Faculty of Pharmacy, Medical Academy, Lithuanian University of Health Sciences, Sukileliu pr. 13, LT-50161 Kaunas, Lithuania

**Keywords:** multiple emulsion, microbiological challenge test, EpiDerm™, patch test, skin irritation, phototoxicity

## Abstract

The multiple W/O/W emulsion supplemented with the extracts of *Rosmarinus officinalis* L., *Avena sativa* L. and *Linum usitatissimum* L. was prepared in the study, its active compounds were determined by HPLC and its safety was evaluated in vitro by the means of reconstituted human skin model EpiDerm™ for the assessment of its irritation, phototoxicity and early skin inflammation effects and by the 48 h human skin patch test for its skin irritation and allergenic potential. The microbiological challenge test of W/O/W emulsion was performed to ensure its preservation efficiency. The results showed that the W/O/W emulsion loaded with self-preserving plant-based bio-actives had no irritant potential, was not phototoxic and did not provoke skin inflammation or sensitization and thus could be used as a safe base for cosmetic products. Furthermore, our results demonstrate that the safety evaluation of cosmetic ingredients of natural or organic origin could be easily performed using reconstructed human skin model EpiDerm™ similar to the well-defined chemicals used in the cosmetics industry.

## 1. Introduction

In recent years, plant-derived substances have been widely investigated not only as bioactive ingredients, but also as the additives in the production of the semi-solid pharmaceuticals and cosmetics to enhance their moisturizing properties, decrease oxidative and inflammatory processes in the skin and to improve their characteristics as surfactants or viscosity modifiers [1]. Their versatile properties determine the variety of possible applications they could be used in ecological cosmetics, though their technological impact has been often overlooked. Water-in oil-in water (W/O/W) multiple emulsions are composed of small aqueous phase droplets which are trapped inside larger oil droplets dispersed in a continuous phase. These complex emulsions have been in the focus of attention as innovative vehicles for the drug delivery, especially in cosmetics and pharmaceutical products due to the various functional properties such as masking of odor and taste, protection against oxidation to prolong the shelf-life of bioactives. Multiple emulsions also have potential as micro carriers of the lipophilic and hydrophilic ingredients entrapped in the inner phase, which can be subsequently released [2,3]. Generally, W/O/W or O/W/O are prepared by a two stage or one stage emulsification process, thought the loss of the internal phase during the inversion or shear stress and the complexity of the techniques are the major issues [2,4] Recently, we designed the novel water-in oil-in water (W/O/W) multiple emulsion which was formed directly by using *Rosmarinus officinalis* L. extract and it was stabilized with natural structuring agents like *Avena sativa* L. and *Linum usitatissimum* L. improving its rheological properties and skin characteristics [5,6].

However, the plant-based bioactive compounds may pose the risk of skin sensitization and allergic reactions [7] and therefore, the safety of natural components in cosmetic and pharmaceutical products should be carefully evaluated to prevent the aggravation of existing skin conditions during their application [1]. It has been reported that ingredients of natural origin such as argan oil, wolf’s bane, lavender, peppermint and some essential oils may cause contact dermatitis and urticaria [8,9,10]. However, these ingredients are widely used in cosmetics and pharmaceutical preparations and the evidence of their safety is underreported. The protein-containing crops, loaded in the W/O/W emulsion, also may induce an allergic reaction [11,12]. Therefore, the toxicological assessment of topically applied products containing known sensitizing agents should be mandatory.

The EU Regulation (EC) N° 1223/2009 foresees that the safety assessment of the finished product must be performed before it can be placed on the EU market, moreover, the testing of the finished cosmetic products and cosmetic ingredients on animals is prohibited [13]. Previously, the potential of a compound or the preparation to cause the skin irritation or corrosion has been evaluated using a rabbit skin test [14]. The public organizations and relevant institutions are requiring minimization of the in vivo skin corrosion testing on animals due to the pain and trauma it causes to the test subjects [15]. An alternative strategy to the animal test was the use of human volunteers to identify skin irritation hazard [16,17]. To comply with these requirements, we have used alternative toxicological assays in this study. Recently, the reconstituted 3D human skin models were validated to identify the chemical sensitization in vitro [13].They are applicable to cosmetic, pharmaceutical and medical device safety testing [18]. In comparison to the epidermis models or the single-layer cell cultures, the 3D models have better performance in the efficacy testing due to the similarity to human skin especially the interaction between the dermis and epidermis. It serves as a more acceptable instrument for the formulation chemists, because not only a single chemical ingredient but also their composition can be tested. The repeated application process and the long term incubation can be performed as well, which is extremely important in the safety assessment of the products [19]. However, despite the wide acceptance of these models for the regulatory purposes, they are rarely used to test cosmetic products containing natural ingredients. There is an increasing evidence that plant-derived materials safely used many years for food or medicine production might cause allergic reactions and increase health risks to the consumers when used in the cosmetic industry [20,21]. Nevertheless, a thorough safety assessment and clinical scientific data of botanicals in cosmetics is often overlooked [21]. Therefore, this study is one of the first attempts to evaluate the microbiological and toxicological safety of the innovative self-preserving multiple emulsion with natural ingredients. A microbiological challenge test was used to evaluate preservation efficiency of the W/O/W emulsion and reconstituted human skin model EpiDerm™ [13,22]—to evaluate its irritation [22], phototoxicity [23] and early skin inflammation effects. The skin sensitization of the multiple emulsion was assessed based on the 48 h human skin patch test [24].

## 2. Materials and Methods

### 2.1. Materials

Dried rosemary (*Rosmarinus officinalis* L.) leaves and flaxseed (*Linum usitatissimum* Linn.) were purchased from UAB “Sirdazole”, Lithuania. The oat groats (*Avena sativa* L.) used for the preparation of colloidal oatmeal were obtained from UAB “Skaneja”, Lithuania. The viscosity modifier ViscOptima^TM^ SE (Croda, East Yorkshire, UK), consisting of Sodium Polyacrylate, Ethylhexyl Cocoate, PPG-3 Benzyl Ether Myristate and Polysorbate 20, was used as an emulsifying agent. Extra-virgin olive oil was purchased from UAB “Anira”, Lithuania. Ethanol (96%) was purchased from UAB “Stumbras” (Kaunas, Lithuania). Distilled water was used throughout the experiment.

The EpiDerm^TM^ (EPI-200) skin inserts, the three-dimensional reconstructed human skin model consisting of normal human-derived keratinocytes, multiple viable cell layers and functional stratum corneum and MTT Kit (MTT-100) to test cell viability were from MatTek In Vitro Life Science Laboratories Corporation (Bratislava, Slovakia). The Quantikine^®^ Human IL-1α/IL-1F1 immunoassay was from R&D Systems (Minneapolis, MN, USA).

### 2.2. Preparation of Plant Extracts

Dried rosemary leave extract was prepared by the ultrasound assisted extraction (Bandelin electronic GmbH & Co.KG, Berlin, Germany) using ethanol (90%) as the solvent. The solvent to material ratio was 1:15, the extraction temperature was 60 °C; the extraction time was 10 min. The HPLC analysis showed that the extract contained 0.58 mg/mL rosmarinic acid, 1.06 mg/mL ursolic acid and 0.62 mg/mL oleanolic acid [5].

Colloidal oatmeal was prepared by the extraction of oats carried out by using magnetic stirrer (MSH-20A, Witeg Labotechnik GmbH, Wertheim, Germany). Whole oat groats were extracted in 250 mL flask for 1 h at 98 ± 2 °C temperature, using distilled water as the extraction solvent. Solvent to material ratio was 1:20. The hot extract was filtered through four layers of cheesecloth and was allowed to cool to room temperature. According to the TLC analysis it contained β-glucan (0.44 ± 0.02%), l-(+)-arabinose (340.44 ± 0.01 mg/g dried preparation), d-(+)-xylose (12.64 ± 0.01 mg/g dried preparation), d-(+)-galacturonic acid (34.21 ± 0.04 mg/g dried preparation) [5].

The flaxseed mucilage extraction was performed with boiling water at 1:20 ratio for 60 min using magnetic stirrer (MSH-20A, Witeg Labotechnik GmbH, Wertheim, Germany). The hot mixture was immediately filtered through the cotton based cheesecloth and left to cool down to the room temperature. The mucilage was examined by TLC [5]. It contained 1-l-rhamnose monohydrate (11.01 ± 0.01 mg/g dried extract), l-(+)-arabinose (165.72 ± 0.01 mg/g dried extract), D-(+)-galactose (50.72 ± 0.01 mg/g dried extract), d-(+)-xylose (297.76 ± 0.01 mg/g dried extract), D-(+)-galacturonic acid (21.46 ± 0.04 mg/g dried extract).

The conditions of the HPLC and TLC are published in the previous studies [5,25].

### 2.3. Preparation of the W/O/W Emulsion

The optimal composition of the W/O/W emulsion was determined in our previous study using the experimental mixture design matrix (Design Expert 9.0.4.01, Stat- Easy Inc., Minneapolis, MN, USA) [26]. Briefly, 0.5% of emulsifier ViscOptima^TM^ SE was mixed into the olive oil (20%), followed by the addition of continuous phase containing water (3.79%), rosemary extract (7.5%), flaxseed mucilage (24.18%) and colloidal oatmeal (44.03%). The optimal technological parameters were previously determined, so the emulsion was stirred with mechanical stirrer IKA Eurostar 200 digital (IKA^®^-Werke GmbH & Co. KG, Staufen, Germany) at a speed of 800 rpm for 15 min [5]. The emulsion was stable as determined by 28 days short term stability assay and accelerated stability assay (at 38 ± 2 °C and 75 ± 5% relative humidity) [5,27,28].

### 2.4. Microbiological Challenge Testing

The experiment was carried out according to the ISO 11930:2012 at room temperature (20 ± 3 °C). The test strains were *Aspergillus brasiliensis* (ATCC 16404), *Candida albicans* (ATCC 10231), *Escherichia coli* (ATCC 8739), *Staphylococcus aureus* (ATCC 6538) and *Pseudomonas aeruginosa* (ATCC 9027). The culture media was tryptic soy agar (TSA) for bacteria, sabourad dextrose agar medium (SDA) for *C. albicans* and potato dextrose agar (PDA) for *A. brasiliensis*. The incubation temperature of Petri dishes was 32.5 ± 2.5 °C for 48–72 h for bacteria and *C. albicans* and 22.5 ± 2.5 °C for 3–5 days for *A. brasiliensis.* For each stain 20 g of cream sample was used. 0.2 mL of calibrated inoculum was added. 1.0 × 10^5^ ÷ 1.0 × 10^6^ cfu/mL for bacteria and 1.0 × 10^4^ ÷ 1.0 × 10^5^ cfu/mL for fungi (Table 1). The samples were successfully neutralized with LT 100 liquid broth and the growth of the bacteria was observed after 7, 14 and 28 days.

### 2.5. In Vitro Skin Irritation Test

The validated ET-50 protocol provided by the supplier and was used for the in vitro skin irritation test. The EpiDerm^TM^ skin inserts were transferred to 6-well plates containing 0.9 mL Dulbecco’s Modified Eagle’s Medium (DMEM) per well and pre-incubated in the humidified 37 °C, 5% CO_2_ incubator overnight. Before the test, the assay medium was replaced by the fresh medium and 100 µL of test sample was applied topically on the EpiDerm^TM^ skin inserts and the samples were incubated for 3, 5 and 18 h. 1% Triton X-100 was used as positive control while distilled water as a negative control. Three replicates of EpiDerm^TM^ skin inserts were used for each experiment.

At the end of each exposure time, the assay media from the 6-well plates were collected and used further for the IL-1α assay. The EpiDerm^TM^ skin inserts were rinsed with PBS twice and transferred to 3-(4,5-Dimethylthiazol-2-yl)-2,5-diphenyltetrazolium bromide (MTT; 300 µL of a 0.3 mg/mL solution) containing 24-well plate for the cell viability testing. For the MTT test, the samples were kept in a 5% CO_2_ environment for 3 h. 2 mL of isopropanol was used to extract formazan. The optical density (OD) of 200 µL of the isopropanol extract was measured at 570 nm using a UV-Vis Spectrophotometer. The test material was considered to be irritant to skin if the tissue viability after exposure was ≤ 50%.

### 2.6. In Vitro Skin Phototoxicity Test

The pre-validated phototoxicity protocol was provided by the supplier and was used for the in vitro skin phototoxicity test. The EpiDerm^TM^ skin inserts were treated initially as in the skin irritation assay. For the exposure, 5 concentrations of the test material (dissolved in water) were topically applied onto 2 tissues per concentration, water was used as negative control. The second 12 tissue set was treated similarly. After the incubation of 18 h the half of the tissue samples were exposed to 1.7 mW/cm^2^ UVA and the others were kept in the dark for the same amount of time. Further on, they were washed with PBS, transferred to the fresh medium and incubated for 18 h. Then, the assay medium was replaced by the MTT-medium and the samples were incubated for 3 h. Finally, PBS was used to wash the inserts, the formazan was extracted with isopropanol. The OD was measured by the plate spectrophotometer at 570 nm and the cell viability was expressed as % of the corresponding negative control (irradiated or non-irradiated). For the positive control, the parallel experiment with the known phototoxic agent—0.1% chlorpromazine solution (diluted to 5 concentrations) was performed.

### 2.7. IL-1α Assay for SKIN inflammation Testing

The IL-1α content was assessed using the Quantikine^®^ Human IL-1α/IL-1F1 immunoassay kit according to the DLA-50 protocol given by the manufacturer. 200 µL of the tissue media collected after 3, 5 and 18 h exposure during the in vitro skin irritation test as well as the positive (1% Triton X-100) and the negative controls (distilled water) and 50 µL of assay diluent were added to the antibody-precoated microplate wells and incubated for 2 h at the room temperature. Afterwards the streptavidin-horse radish peroxidase-conjugated secondary antibodies were added to the pre-washed test samples and the plate was incubated for 60 min at 22 ± 2 °C. Then, the substrate solution was added and the plate was incubated again for 20 min. The chemical reaction was stopped by adding 50 µL of 2 N sulfuric acid. The OD was measured by the microplate reader at 450 nm with a correction wavelength of 570 nm. The standard curve was used to calculate the concentration (R = 0.99864). IL-1α concentration higher than 85 pg/mL was considered a marker of inflammation.

### 2.8. 48 Hours Human Skin Patch Test

The study was carried out at the clinic of general and aesthetic dermatology “Clinic In” (Vilnius, Lithuania). The test methods and the selection procedure of human volunteer were executed according to the Declaration of Helsinki and International Ethical Guidelines for Biomedical Research Involving Human Subjects [29].

A total of 18 healthy volunteers (6 men and 12 women) above 18 years old were selected. They have signed informed consent form before the beginning of the study. The age of participants varied between 24 and 71 years (M = 44.7 years). The selected criteria for inclusion were: healthy upper back skin with the absence of dermatological conditions, inflammation or dryness which resulted in desquamation; no participation in other medical studies; no known allergies and no application of topical drugs or the use of oral drugs that may affect skin reactions. The exclusion criteria were: an active skin and other uncontrollable disease, the use of medicine that might provoke skin reactions, pregnancy and breastfeeding, the age younger than 18 years old, Lithuanian language knowledge not sufficient to understand the information provided. The investigation was carried out according to the COLIPA recommendations and the Regulation (EC) No 1223/2009 of the European Parliament and of the Council of 30 November 2009 on cosmetic products [13].

The participants 12 h before and during the study could not use any skin care products. 20 mg of test material was applied to the Finn chamber (Epitest Ltd. Oy., Tuusula, Finland) on Scanpor tape (Alpharma AS, Norway). The Finn chamber size was 11.0 mm and the size of the aluminum cup was 8.0 mm. On the upper back of volunteers, the chamber strips were placed and sealed for 48 h using surgical tape in order to prevent peeling of adjacent skin and avoid the unnecessary movement of the chambers containing test sample (Mepore, Molnlycke Health Care, Gothenburg, Sweden). The skin around it was marked with a waterproof marker. Participants were assessed by dermatologist and allergologist after 48, 72, 96 h and 7 days according to the criteria set out by the International Contact Dermatitis Research Group [30,31].

### 2.9. Statistical Analysis

The results are given as mean ± SD. One-way and two-way ANOVA with Dunnett’s post hoc test was used to carry out the statistical analysis (Prism v. 5.04, GraphPad Software Inc., La Jolla, CA, USA). The level of significance of 95% was chosen for the study.

## 3. Results and Discussion

Topically applied products are subject to a range of testing to protect users from microbiological and chemical contamination and from other possible toxic effects. Usually, eco-friendly or natural ingredients-based products are more susceptible to microbiological contamination and they have shorter shelf life unless heavily preserved. However, some natural ingredients possess antimicrobial properties and their incorporation in the formulation helps to decrease the amount of chemical preservatives or even replace them completely [32,33].

Since the composition of created innovative multiple emulsion is natural-based and preservative free and according to the microbiological quality assay carried out in our previous study the CFU complies with the requirements of European Pharmacopoeia 7.0 01/2011:50104, the possible microbial contamination during the usage period must be still considered before the in vivo and in vitro evaluation to avoid the possible negative reactions. To assess the robustness of the products against microbial contamination, the challenge test according to the ISO 11930:2012 was carried out. The W/O/W emulsion contains rosemary extract which is widely used in the cosmetics and food industry due to the antimicrobial properties [34,35,36]. Moreno et al. have determined that main active compounds responsible for inhibiting bacterial growth are phenolic acids: rosmarinic and carnosic acids [37]. Therefore, the rosmarinic acid was identified in the extract which was used in the formulation of the self-preserving multiple emulsion based on the hypothesis that the preservation efficiency of the product is in line with the requirements of Regulation (EC) No 1223/2009. The obtained data confirmed the hypothesis that the W/O/W emulsion is efficiently preserved using herbal extracts (Table 2). The growth of the bacteria and fungi was not observed after 7, 14 and 28 days. Based on the literature and our experimental data it could be concluded that rosemary extract and/or its essential oils are suitable as an alternative to chemical preservatives for the topical semi-solid preparations [35,38]. The microbiological quality of the multiple emulsion complies with the requirements of the regulatory documents and the multiple emulsion is safe to use in further in vitro and in vivo studies.

**Figure 1:**
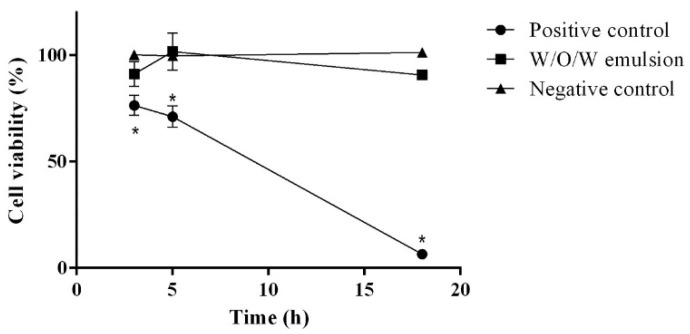
The influence of W/O/W emulsion on the cell viability during in vitro skin irritation test. N = 4 * *p* < 0.05 vs. negative control and W/O/W emulsion.

To proceed further with the development of the emulsion-based cosmetic product containing herbal extracts we assessed its safety using the reconstituted 3D human skin models as an alternative to the in vivo animal testing. First, the skin irritation potential was evaluated. It was determined that the 0.1% triton X-100 solution (positive control) has a significant impact on the cell viability: after 3 h it decreased by 23.51 ± 4.68%, after 5 h 28.83 ± 5.08% and after 18 h 93.41 ± 0.68% (Figure 1).

**Figure 2:**
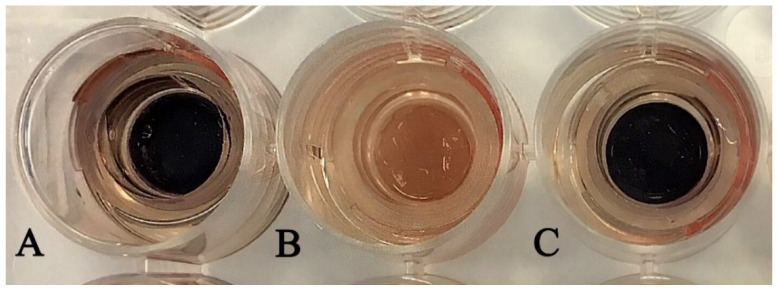
The view of the cell tissues during the in vitro skin irritation test after 18 h of incubation and cell viability determination by MTT. (**A**)—negative control, (**B**)—positive control, (**C**)—W/O/W emulsion.

The concentration of ethanolic rosemary extract in the composition of W/O/W emulsion was 7.5%. According to the Food and Drug Administration (USA) the recommended concentration of the rosemary extract in the topical products should be 0.001–10%, as these concentrations did not result in skin irritation [39]. These data comply with the results of our study: the application of the W/O/W emulsion did not change the cell viability which varied between 90.74% and 101.71% (*p* > 0.05). The influence of the presence of ethanol in the topically applied products on the skin erythema was widely investigated in various scientific studies, thus, in our results it is worth mentioning that there was no statistically significant difference in the cell viability between the negative control (distilled water) and multiple emulsion, which contains ethanol (Figure 2). The obtained results corresponded with the data obtained by the recent studies that ethanol-based products were well tolerated by the skin and could decrease the skin moisture only insignificantly [40,41].

**Figure 3:**
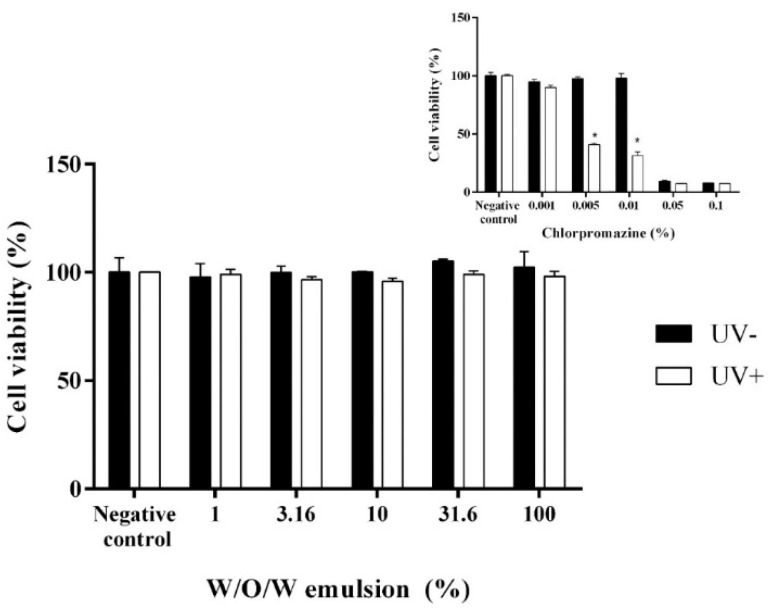
The influence of W/O/W emulsion and chlorpromazine on the cell viability during in vitro phototoxicity test. N = 4 * *p* < 0.05 vs. UV−.

In the skin phototoxicity test the EpiDerm^TM^ skin inserts were exposed to 5 different concentrations of the water-diluted multiple W/O/W emulsions loaded with the extracts of *Rosmarinus officinalis* L., *Avena sativa* L. and *Linum usitatissimum* L in order to determine the threshold of photosensitivity. The parallel experiment with known phototoxic and cytotoxic compound chlorpromazine was performed as a positive control to prove the efficiency of the selected method [42]. It is already known that 0.005% and higher concentrations of chlorpromazine have phototoxic effect. 0.05% and higher concentrations of chlorpromazine results in the cytotoxicity when applied on the skin [43,44]. Nobile et al. have determined that *R. officinalis* and *C. paradise* extracts had photo-protective properties and could prevent the negative reactions caused by the UV rays when used topically on the skin [45]. The results of our study correspond with the data presented by other researchers. We found that the cell viability of the tissues affected with test sample (the W/O/W emulsion which contains herbal extracts of rosemary, oats and flaxseed) varied between 95.77 and 105.28%. The concentration of the W/O/W emulsion had no significant impact on the cell viability as well [Figure 3]. No difference in the cell viability was observed between the test samples and negative control (*p* < 0.05). As expected, the positive control (chlorpromazine) decreased the cell viability by 59.24–68.67% at a concentration range of 0.005–0.01% when exposed to UV (*p* < 0.05 vs. negative control). At 0.05% and higher concentrations of chlorpromazine decreased the cell viability of the EpiDerm tissues by more than 90% not depending on the UV exposure. Thus, our results showed that the tested multiple W/O/W emulsion had neither phototoxic nor cytotoxic effects.

**Figure 4:**
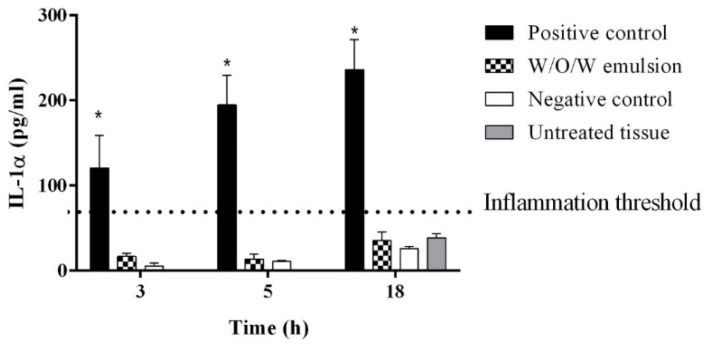
The influence of W/O/W emulsion on the inflammation marker IL-1α release in vitro. N = 4 * *p* < 0.05 vs. negative control, W/O/W emulsion and untreated tissue.

A lot of studies have evaluated and determined the antioxidant and anti-inflammatory effect of rosemary and its constituents [46,47]. The oatmeal extract containing β-glucans and active ingredients of the flaxseed extract are mentioned in the various scientific sources as natural ingredients with the potential to suppress inflammation as well [48,49]. Therefore, during the determination of the early inflammation in vitro the results showed that the amount of released IL-1α into the media by the tissues with the W/O/W emulsion did not differ significantly from the negative control and untreated tissues, the IL-1α concentrations were below 85 pg/mL (Figure 4). It was concluded that the application of the multiple emulsion with botanical extracts did not cause any inflammation in human keratinocytes.

Recently, the research on botanical extracts and their active ingredients in food and cosmetic industry opened-up a pathway for the new applications of these bioactives, thus demanding a check to determine the frequency of allergic reactions [20,21]. In order to avoid the allergic reactions of the W/O/W emulsion which was formed using rosemary, flaxseed and oat extracts, the safety of the product was evaluated not only by the in vitro study of the 3D reconstructed human epidermis model, but also by the human skin patch testing. The total of 18 healthy volunteers have participated in the study. The results were evaluated after 48, 72, 96 h and 7 days after the W/O/W emulsion application using the Finn chambers. Despite that several studies have demonstrated that 0.1% rosemary leaf extract applied topically could cause skin erythema, our data contradicted with their results [50,51]. Moreover, Monice et al. stated that it is safe to use rosemary leaf extract up to the 10 mL/kg and the pretreatment with 10 to 1000 µg/cm^2^ of the rosemary extract could significantly reduce ear edema caused by 25 ng/cm^2^ of 12-tetradecanoylphorbol-13-acetate in mice [52]. All the participants of the current study had negative reaction according to the International Contact Dermatitis Research Group criteria. Even if there are some contradictions related to the herbal extract (such as rosemary and oat)-induced allergic reactions, our results obtained by a patch test complies with the data obtained by the 3D reconstructed human epidermis in vitro, verifying that the innovative W/O/W emulsion does not cause any skin irritation in vivo.

## 4. Conclusions

Due to the diverse phytochemical composition of botanical extracts which are used as the functional or active ingredients in topically applied formulations it is pertinent to evaluate their safety to avoid the possible allergic/irritation reactions. The 3D reconstructed human epidermis models can be used as an alternative to animal testing that allows to predict the skin reaction and correlates with the results of in vivo testing. According to the obtained data, it could be concluded that the object of this study—the innovative multiple W/O/W type emulsion formed and preserved directly using the plant extracts complies with the safety requirements and may be used as an innovative base in the development of the final high-quality cosmetic or pharmaceutical products. In the future, further experiments are foreseen to evaluate the possible use of rosemary and other extracts involved in the study as natural preservatives as well as to evaluate the efficacy of these complex emulsions on the human skin in vivo.

## Figures and Tables

**Table 1:** Demonstration of the neutralizer efficacy.

Microorganisms	N, cfu/mL	N_0_, cfu/mL	Nvf, cfu/mL	Nvn, cfu/mL	Nvf ≥ 0.5 Nvn	Nv, cfu/mL
*Escherichia coli*	3.2 × 10^7^	3.2 × 10^5^	2.5 × 10^2^	2.6 × 10^2^	>0.5	3.0 × 10^2^
*Staphylococcus aureus*	2.9 × 10^7^	2.9 × 10^5^	2.0 × 10^2^	2.1 × 10^2^	>0.5	2.9 × 10^2^
*Pseudomonas aeruginosa*	3.7 × 10^7^	3.7 × 10^5^	1.2 × 10^2^	1.4 × 10^2^	>0.5	1.2 × 10^2^
*Candida albicans*	1.4 × 10^6^	1.4 × 10^4^	1.1 × 10^2^	9.5 × 10^1^	>0.5	1.0 × 10^2^
*Aspergillus brasiliensis*	1.0 × 10^6^	1.0 × 10^4^	8.0 × 10^1^	5.9 × 10^1^	>0.5	7.2 × 10^1^

N—quantity of the initial numbers of microorganisms; N_0_—N/100; Nvf—number of microorganisms present in the test mixture with the neutralizer and formulation; Nvn—number of microorganisms present in the test mixture with the neutralizer in the absence of formulation; Nv—inoculum control.

**Table 2:** Results of a microbiological challenge testing (efficacy of the antimicrobial protection).

Microorganisms	Log Reduction Values (R_x_ = lgN_0_ − lgN_x_)					
	T7	Criteria	T14	Criteria	T28	Criteria
*Escherichia coli*	4.5	≥3	4.5	≥3 and NI	4.5	≥3 and NI
*Staphylococcus aureus*	4.5	≥3	4.5	≥3 and NI	4.5	≥3 and NI
*Pseudomonas aeruginosa*	4.6	≥3	4.6	≥3 and NI	4.6	≥3 and NI
*Candida albicans*	3.1	≥1	3.1	≥1 and NI	3.1	≥1 and NI
*Aspergillus brasiliensis*	3.0	-	3.0	≥0	3.0	≥0

N_0_—number of microorganisms inoculated at time t_0_; N_x_—number of surviving microorganisms at each sampling time t_x_ (T7, T14, T28); NI—no increase in the count from the previous contact time T7, T14, T28 days.

## Data Availability

Not applicable.
